# *N*-[(*E*)-4-Fluoro­benzyl­idene]-3,4-di­methyl­aniline

**DOI:** 10.1107/S1600536812030474

**Published:** 2012-07-10

**Authors:** Yue-Bao Jin, Yong-Kang Chang, Ying Zhang, Ke-Wei Lei

**Affiliations:** a State Key Laboratory Base of Novel Functional Materials and Preparation Science, Institute of Solid Materials Chemistry, Faculty of Materials Science and Chemical Engineering, Ningbo University, Ningbo 315211, People’s Republic of China

## Abstract

In the title Schiff base, C_15_H_14_FN, the N=C bond length of 1.263 (2) Å is shorter than the N—C bond [1.426 (2) Å], indicating a typical imine double bond. Moreover, the C—N—C angle is 118.5 (2)°. The benzene rings form a dihedral angle of 51.22 (5)°.

## Related literature

For general background on the use of Schiff bases as ligands in inorganic and organometallic chemistry, see: Xia *et al.* (2009 ▶); Harries & Orford (1983 ▶); Rodriguez de Barbarin *et al.* (1994 ▶). For similar structures, see: Xia *et al.* (2009 ▶); Lindeman *et al.* (1981 ▶). For a related synthetic procedure, see: Chen *et al.* (2005 ▶).
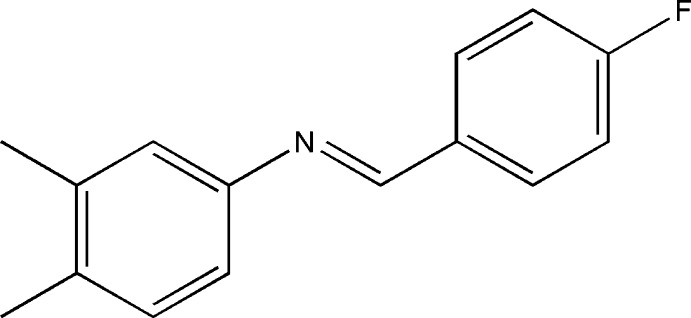


## Experimental

### 

#### Crystal data

C_15_H_14_FN
*M* *_r_* = 227.27
Orthorhombic, 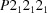

*a* = 7.7487 (3) Å
*b* = 11.3404 (3) Å
*c* = 14.2969 (4) Å
*V* = 1256.31 (7) Å^3^
*Z* = 4
Mo *K*α radiation
μ = 0.08 mm^−1^
*T* = 293 K
0.42 × 0.21 × 0.16 mm

#### Data collection

Rigaku R-AXIS RAPID diffractometer
Absorption correction: multi-scan (*ABSCOR*; Higashi, 1995 ▶) *T*_min_ = 0.978, *T*_max_ = 0.988
10903 measured reflections
1941 independent reflections
1476 reflections with *I* > 2σ(*I*)
*R*_int_ = 0.023

#### Refinement

*R*[*F* ^2^ > 2σ(*F* ^2^)] = 0.040
*wR*(*F* ^2^) = 0.103
*S* = 1.05
1941 reflections
154 parameters
H-atom parameters constrained
Δρ_max_ = 0.10 e Å^−3^
Δρ_min_ = −0.15 e Å^−3^

### 

Data collection: *RAPID-AUTO* (Rigaku, 1998 ▶); cell refinement: *RAPID-AUTO*; data reduction: *CrystalStructure* (Rigaku/MSC, 2004 ▶); program(s) used to solve structure: *SHELXS97* (Sheldrick, 2008 ▶); program(s) used to refine structure: *SHELXL97* (Sheldrick, 2008 ▶); molecular graphics: *SHELXTL* (Sheldrick, 2008 ▶); software used to prepare material for publication: *SHELXL97*.

## Supplementary Material

Crystal structure: contains datablock(s) global, I. DOI: 10.1107/S1600536812030474/im2381sup1.cif

Structure factors: contains datablock(s) I. DOI: 10.1107/S1600536812030474/im2381Isup2.hkl

Supplementary material file. DOI: 10.1107/S1600536812030474/im2381Isup3.cml

Additional supplementary materials:  crystallographic information; 3D view; checkCIF report

## Figures and Tables

**Fig. 1.**
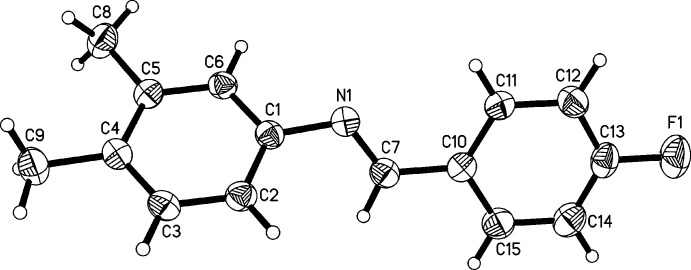
Molecular structure of the title compound showing displacement ellipsoids on the 30% probability level.

## supplementary crystallographic information

### Comment

Schiff bases are among the most useful ligands in coordination chemistry as they readily form stable complexes with most transition metals (Xia *et al.*, 2009; Harries & Orford, 1983; Rodriguez de Barbarin *et al.*, 1994).

The molecular structure of the title compound is illustrated in Fig. 1. Bond angles and bond lengths are within normal ranges. The F1—C13 bond length is 1.361 (2) Å. The N1=C7 bond length of 1.263 (2) Å is shorter than the N1—C1 bond [1.426 (2) Å], indicating a typical imine double bond. Moreover, the C1—N1—C7 bond angle is 118.5 (2)°. The two benzene rings form a dihedral angle of 51.22 (5)° (Xia *et al.*, 2009; Lindeman *et al.*, 1981).

### Experimental

4-Fluorobenzaldehyde (20 mmol, 2.48 g) and 3,4-dimethylbenzenamine (20 mmol, 2.42 g) were dissolved in ethanol and the solution was refluxed for 1 h in a round bottom flask according to a procedure of Chen *et al.* (2005). After evaporation of the solvent the crude product was recrystallized twice from methanol to give a pure yellow product (yield: 82.5%). Elemental analysis calcd. for C_15_H_14_FN: C, 79.29; H, 6.21; N, 6.16; Found: C, 79.25; H, 6.22; N, 6.18%.

### Refinement

All H atoms were placed in geometrically idealized positions and constrained to ride on their parent atoms (C—H = 0.93 Å) and *U*_iso_(H) values equal to 1.2 *U*_eq_(C).

### Crystal data

**Table:**

C_15_H_14_FN	*F*(000) = 480
*M**_r_* = 227.27	*D*_x_ = 1.202 Mg m^−^^3^
Orthorhombic, *P*2_1_2_1_2_1_	Mo *K*α radiation, λ = 0.71073 Å
Hall symbol: P 2ac 2ab	Cell parameters from 4863 reflections
*a* = 7.7487 (3) Å	θ = 1.0–29.1°
*b* = 11.3404 (3) Å	µ = 0.08 mm^−^^1^
*c* = 14.2969 (4) Å	*T* = 293 K
*V* = 1256.31 (7) Å^3^	Block, yellow
*Z* = 4	0.42 × 0.21 × 0.16 mm

### Data collection

**Table:**

Rigaku R-AXIS RAPID diffractometer	1941 independent reflections
Radiation source: fine-focus sealed tube	1476 reflections with *I* > 2σ(*I*)
Graphite monochromator	*R*_int_ = 0.023
ω scans	θ_max_ = 29.1°, θ_min_ = 3.0°
Absorption correction: multi-scan (*ABSCOR*; Higashi, 1995)	*h* = −9→10
*T*_min_ = 0.978, *T*_max_ = 0.988	*k* = −14→14
10903 measured reflections	*l* = −18→18

### Refinement

**Table:**

Refinement on *F*^2^	Primary atom site location: structure-invariant direct methods
Least-squares matrix: full	Secondary atom site location: difference Fourier map
*R*[*F*^2^ > 2σ(*F*^2^)] = 0.040	Hydrogen site location: inferred from neighbouring sites
*wR*(*F*^2^) = 0.103	H-atom parameters constrained
*S* = 1.05	*w* = 1/[σ^2^(*F*_o_^2^) + (0.0515*P*)^2^ + 0.1023*P*] where *P* = (*F*_o_^2^ + 2*F*_c_^2^)/3
1941 reflections	(Δ/σ)_max_ = 0.001
154 parameters	Δρ_max_ = 0.10 e Å^−^^3^
0 restraints	Δρ_min_ = −0.15 e Å^−^^3^

### Special details

**Table:**

Geometry. All e.s.d.'s (except the e.s.d. in the dihedral angle between two l.s. planes) are estimated using the full covariance matrix. The cell e.s.d.'s are taken into account individually in the estimation of e.s.d.'s in distances, angles and torsion angles; correlations between e.s.d.'s in cell parameters are only used when they are defined by crystal symmetry. An approximate (isotropic) treatment of cell e.s.d.'s is used for estimating e.s.d.'s involving l.s. planes.
Refinement. Refinement of *F*^2^ against ALL reflections. The weighted *R*-factor *wR* and goodness of fit *S* are based on *F*^2^, conventional *R*-factors *R* are based on *F*, with *F* set to zero for negative *F*^2^. The threshold expression of *F*^2^ > σ(*F*^2^) is used only for calculating *R*-factors(gt) *etc*. and is not relevant to the choice of reflections for refinement. *R*-factors based on *F*^2^ are statistically about twice as large as those based on *F*, and *R*- factors based on ALL data will be even larger.

### Fractional atomic coordinates and isotropic or equivalent isotropic displacement parameters (Å^2^)

**Table:**

	*x*	*y*	*z*	*U*_iso_*/*U*_eq_	
N1	0.5157 (2)	0.33118 (14)	0.66820 (10)	0.0529 (4)	
F1	0.3419 (2)	0.37162 (14)	1.10226 (9)	0.0933 (5)	
C6	0.5091 (2)	0.27263 (16)	0.50536 (13)	0.0487 (4)	
H3A	0.4576	0.2024	0.5241	0.058*	
C1	0.5509 (2)	0.35665 (15)	0.57238 (12)	0.0472 (4)	
C10	0.4210 (2)	0.39849 (16)	0.81944 (13)	0.0502 (4)	
C4	0.6225 (2)	0.39542 (17)	0.38272 (12)	0.0503 (4)	
C12	0.4505 (3)	0.28949 (19)	0.96341 (15)	0.0638 (5)	
H7A	0.4877	0.2234	0.9962	0.077*	
C7	0.4517 (2)	0.41157 (16)	0.71858 (13)	0.0517 (4)	
H8A	0.4225	0.4827	0.6904	0.062*	
C5	0.5424 (2)	0.29105 (16)	0.41079 (12)	0.0488 (4)	
C15	0.3412 (3)	0.48874 (18)	0.86852 (14)	0.0636 (5)	
H10A	0.3062	0.5563	0.8368	0.076*	
C2	0.6304 (2)	0.46021 (16)	0.54411 (13)	0.0534 (4)	
H11A	0.6600	0.5173	0.5880	0.064*	
C14	0.3125 (3)	0.4801 (2)	0.96400 (16)	0.0696 (6)	
H12A	0.2571	0.5401	0.9967	0.083*	
C9	0.6641 (3)	0.4191 (2)	0.28139 (13)	0.0715 (6)	
H13A	0.7189	0.4948	0.2758	0.107*	
H13B	0.5596	0.4187	0.2454	0.107*	
H13C	0.7405	0.3591	0.2585	0.107*	
C13	0.3683 (3)	0.3808 (2)	1.00840 (13)	0.0629 (6)	
C11	0.4761 (3)	0.29881 (17)	0.86783 (13)	0.0571 (5)	
H15A	0.5306	0.2380	0.8357	0.068*	
C3	0.6656 (2)	0.47809 (16)	0.45035 (13)	0.0547 (4)	
H16A	0.7197	0.5476	0.4321	0.066*	
C8	0.4925 (3)	0.19754 (19)	0.34034 (14)	0.0686 (6)	
H17A	0.4392	0.1325	0.3721	0.103*	
H17B	0.5939	0.1705	0.3082	0.103*	
H17C	0.4129	0.2303	0.2959	0.103*	

### Atomic displacement parameters (Å^2^)

**Table:**

	*U*^11^	*U*^22^	*U*^33^	*U*^12^	*U*^13^	*U*^23^
N1	0.0567 (9)	0.0536 (8)	0.0484 (8)	−0.0007 (8)	0.0001 (7)	0.0004 (6)
F1	0.1076 (11)	0.1181 (12)	0.0544 (7)	−0.0131 (10)	0.0177 (8)	−0.0034 (7)
C6	0.0467 (9)	0.0448 (9)	0.0546 (10)	−0.0030 (8)	0.0043 (8)	0.0006 (7)
C1	0.0444 (9)	0.0478 (9)	0.0495 (9)	0.0035 (7)	−0.0015 (8)	0.0031 (7)
C10	0.0439 (9)	0.0534 (9)	0.0532 (10)	−0.0025 (8)	−0.0008 (8)	−0.0032 (8)
C4	0.0431 (9)	0.0556 (10)	0.0521 (10)	0.0019 (8)	−0.0011 (8)	0.0055 (8)
C12	0.0736 (13)	0.0594 (11)	0.0584 (11)	−0.0070 (11)	−0.0023 (11)	0.0069 (9)
C7	0.0494 (10)	0.0533 (10)	0.0525 (10)	0.0010 (8)	−0.0048 (8)	0.0036 (8)
C5	0.0407 (9)	0.0535 (10)	0.0521 (10)	0.0003 (8)	0.0012 (8)	−0.0025 (8)
C15	0.0624 (12)	0.0614 (11)	0.0669 (12)	0.0131 (11)	0.0063 (11)	−0.0003 (10)
C2	0.0556 (11)	0.0486 (9)	0.0560 (10)	−0.0047 (8)	−0.0050 (9)	−0.0011 (8)
C14	0.0643 (12)	0.0753 (13)	0.0691 (13)	0.0067 (12)	0.0145 (11)	−0.0122 (11)
C9	0.0788 (14)	0.0785 (14)	0.0573 (12)	−0.0087 (13)	0.0069 (12)	0.0099 (10)
C13	0.0626 (12)	0.0767 (14)	0.0492 (10)	−0.0163 (12)	0.0079 (10)	−0.0067 (9)
C11	0.0664 (12)	0.0501 (9)	0.0548 (10)	−0.0025 (10)	0.0011 (10)	−0.0039 (8)
C3	0.0549 (10)	0.0489 (9)	0.0601 (10)	−0.0072 (9)	−0.0002 (10)	0.0062 (8)
C8	0.0717 (14)	0.0724 (13)	0.0616 (12)	−0.0123 (11)	0.0099 (11)	−0.0150 (10)

### Geometric parameters (Å, º)

**Table:**

N1—C7	1.263 (2)	C7—H8A	0.9300
N1—C1	1.426 (2)	C5—C8	1.513 (3)
F1—C13	1.361 (2)	C15—C14	1.386 (3)
C6—C1	1.390 (3)	C15—H10A	0.9300
C6—C5	1.392 (2)	C2—C3	1.383 (3)
C6—H3A	0.9300	C2—H11A	0.9300
C1—C2	1.386 (2)	C14—C13	1.364 (3)
C10—C15	1.387 (3)	C14—H12A	0.9300
C10—C11	1.392 (3)	C9—H13A	0.9600
C10—C7	1.469 (2)	C9—H13B	0.9600
C4—C3	1.387 (3)	C9—H13C	0.9600
C4—C5	1.395 (2)	C11—H15A	0.9300
C4—C9	1.508 (2)	C3—H16A	0.9300
C12—C13	1.375 (3)	C8—H17A	0.9600
C12—C11	1.385 (3)	C8—H17B	0.9600
C12—H7A	0.9300	C8—H17C	0.9600
			
C7—N1—C1	118.46 (16)	C3—C2—H11A	120.2
C1—C6—C5	121.56 (16)	C1—C2—H11A	120.2
C1—C6—H3A	119.2	C13—C14—C15	117.8 (2)
C5—C6—H3A	119.2	C13—C14—H12A	121.1
C2—C1—C6	118.92 (17)	C15—C14—H12A	121.1
C2—C1—N1	122.44 (16)	C4—C9—H13A	109.5
C6—C1—N1	118.60 (16)	C4—C9—H13B	109.5
C15—C10—C11	118.99 (18)	H13A—C9—H13B	109.5
C15—C10—C7	119.64 (18)	C4—C9—H13C	109.5
C11—C10—C7	121.33 (17)	H13A—C9—H13C	109.5
C3—C4—C5	118.67 (16)	H13B—C9—H13C	109.5
C3—C4—C9	119.82 (17)	F1—C13—C14	118.3 (2)
C5—C4—C9	121.51 (17)	F1—C13—C12	118.2 (2)
C13—C12—C11	118.1 (2)	C14—C13—C12	123.44 (19)
C13—C12—H7A	121.0	C12—C11—C10	120.55 (19)
C11—C12—H7A	121.0	C12—C11—H15A	119.7
N1—C7—C10	123.40 (17)	C10—C11—H15A	119.7
N1—C7—H8A	118.3	C2—C3—C4	121.94 (17)
C10—C7—H8A	118.3	C2—C3—H16A	119.0
C6—C5—C4	119.28 (16)	C4—C3—H16A	119.0
C6—C5—C8	119.61 (17)	C5—C8—H17A	109.5
C4—C5—C8	121.11 (17)	C5—C8—H17B	109.5
C14—C15—C10	121.2 (2)	H17A—C8—H17B	109.5
C14—C15—H10A	119.4	C5—C8—H17C	109.5
C10—C15—H10A	119.4	H17A—C8—H17C	109.5
C3—C2—C1	119.62 (17)	H17B—C8—H17C	109.5
